# Abortion Policy Positions of Federal Legislators Who Received Support From the American College of Obstetricians and Gynecologists, 2012-2022

**DOI:** 10.1001/jamanetworkopen.2023.10405

**Published:** 2023-04-28

**Authors:** Karisma Chhabria, Vineet Raman, Ghazal Aghagoli, Benjamin P. Brown

**Affiliations:** 1 Northwestern University Feinberg School of Medicine, Chicago, Illinois; 2 University of Minnesota Medical School, Minneapolis; 3 The Warren Alpert Medical School of Brown University, Providence, Rhode Island; 4 Department of Obstetrics and Gynecology, The Warren Alpert Medical School of Brown University, Women and Infants Hospital of Rhode Island, Providence, Rhode Island

## Abstract

This cross-sectional study analyzes the abortion policy positions of legislators financially supported by Ob-GynPAC, a political action committee funded by the American College of Obstetricians and Gynecologists (ACOG), between 2012 and 2022.

## Introduction

The American College of Obstetricians and Gynecologists (ACOG) represents more than 60 000 US obstetricians and gynecologists. ACOG affirms that abortion is essential health care and has long stated its opposition to abortion restrictions.^1,2^ A key way in which ACOG advocates for patients and clinicians is through its national political action committee (PAC), Ob-GynPAC, which is funded by ACOG members and the public.^3^ PAC donations are a major factor in determining congressional election outcomes, thereby enabling representatives and senators to pursue their legislative agendas.^4^ Federal legislation, in turn, is a key area in which US abortion policy may be enacted, along with decisions at the local and state levels, and in the executive and judicial branches. In this serial cross-sectional study, we sought to better understand the potential impact of Ob-GynPAC contributions on federal abortion legislation by characterizing the abortion policy positions of legislators supported by Ob-GynPAC between 2012 and 2022.

## Methods

This study followed the Strengthening the Reporting of Observational Studies in Epidemiology (STROBE) reporting guideline. The Brown University institutional review board determined this study was not human participants research, in accordance with 45 CFR § 46, and was therefore exempt from the requirement for informed consent. We extracted contribution data for Ob-GynPAC from the Federal Election Committee website, for dates ranging from January 1, 2012, to June 30, 2022.^5^ Officials in office during this period are likely to have cast votes on legislation and judicial confirmations that have shaped the current abortion policy landscape. All legislators who received Ob-GynPAC support and who were successfully elected to the House of Representatives or the Senate were included in the study. Nonvoting members of Congress were excluded because they did not directly influence federal policy by voting on legislation. See the eAppendix in Supplement 1 for details.

**Figure 1.**
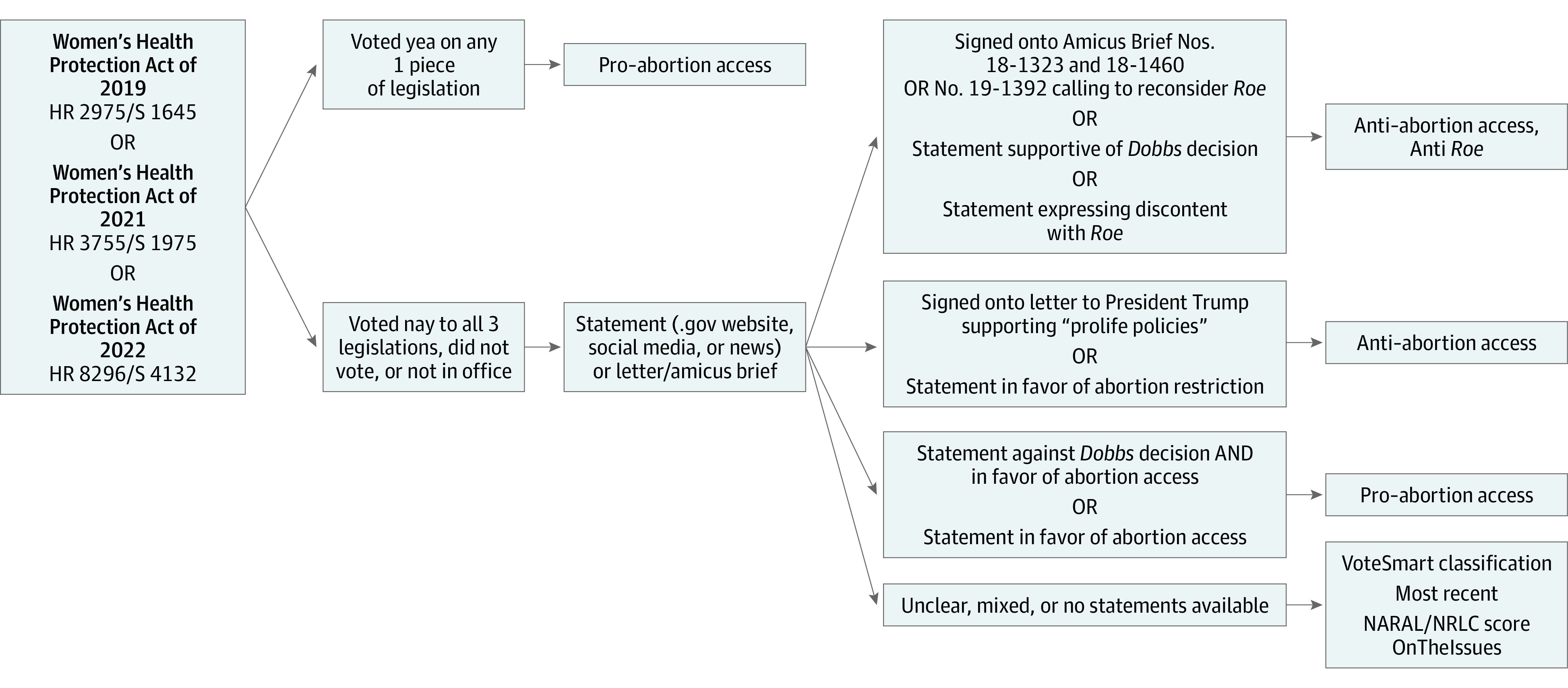
Abortion Policy Position Classification Algorithm Flowchart shows algorithm used to classify legislators’ abortion policy positions. Only votes on the various iterations of the Women’s Health Protection Act were directly evaluated for this study, aggregate scores from organizations including VoteSmart, OnTheIssues, and other advocacy groups accounted for votes on legislation. NARAL indicates National Abortion Rights Action League Pro-Choice America. NRLC indicates National Right to Life Committee.

Our outcome was the abortion policy position of the legislators receiving Ob-GynPAC support. Legislators were classified as anti−abortion access or pro−abortion access on the basis of their public statements and whether their voting history on federal legislation limited or expanded abortion access. We also noted whether a recipient had ever explicitly spoken against *Roe v Wade*^6^ (Figure 1). We analyzed our data with R statistical software, version 4.2.0 (R Project for Statistical Computing).

## Results

Between 2012 and 2022, 294 federal legislators met the inclusion criterion of having received support from Ob-GynPAC. A total of 116 legislators (39.5%) were classified as anti−abortion access, and 70 legislators (23.8%) were also classified as explicitly anti−*Roe v Wade*.^6^ A total of 178 legislators (60.5%) were classified as pro−abortion access. Legislators’ abortion positions remained consistent from 2012 to2022 for all but 5 legislators (1.7%). One senator switched from anti−abortion access to pro−abortion access (0.3%). For legislators who switched positions, we considered the most recent position.

**Figure 2.**
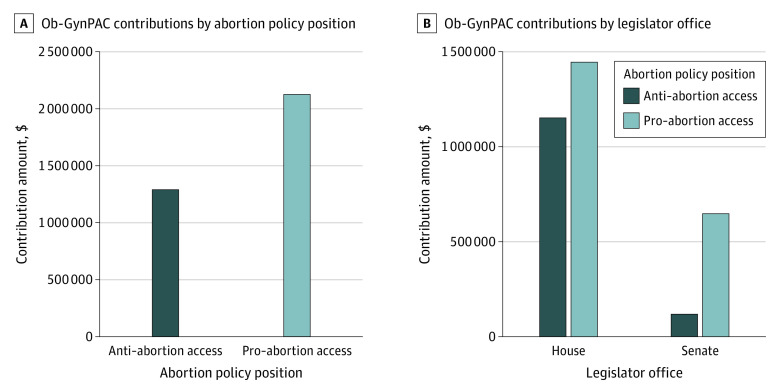
Ob-GynPAC Expenditures, 2012-2022 Graphs show Ob-GynPAC support for individual legislators by abortion policy position (A) and by office (B).

Ob-GynPAC spent a total of $3 363 126 in support of federal legislators. Legislators from the US House of Representatives received $2 597 358 (77.2%), and legislators from the US Senate received $765 768 (22.8%). In total, $1 270 325 (37.8%) went to anti−abortion access legislators, and $2 092 801 (62.2%) to pro−abortion access legislators (Figure 2A). Of the financial support for anti−abortion access legislators, $567 261 (44.7%) went to legislators who explicitly spoke out against *Roe v Wade*.^6^ Ob-GynPAC spent $1 152 025 on anti−abortion access legislators from the US House of Representatives (44.4% of the total support for representatives), and $118 300 on anti−abortion access legislators from the US Senate (15.4% of the total support for senators) (Figure 2B).

## Discussion

A meaningful portion of Ob-GynPAC funding over the last decade ($1 270 325) has gone to support anti−abortion access legislators, likely helping them remain in office, thereby enabling them to pursue their legislative agendas. Our serial cross-sectional study revealed no substantial evidence that an Ob-GynPAC donation was associated with a shift from an anti−abortion access position to a pro−abortion access position. Strengths of our study include the use of a decade of high-quality data, and the use of data from multiple sources to categorize the policy positions of funded legislators. Limitations of our study include the lack of data on state and local donations and on the abortion policy positions of unsuccessful candidates.

Of course, abortion is not the only issue of importance to obstetricians and gynecologists. For example, Medicaid expansion and protecting research funding are also key legislative priorities for ACOG. However, our data indicate that ACOG may be undermining its stated commitment to abortion access and patient autonomy by supporting legislators whose agendas run contrary to the priorities of ACOG.

## Notes

Supplement 1. **eAppendix.** Supplemental Methods
eReferences

Supplement 2.
Data Sharing Statement
