# Supplementary material for: Abortion Policy Positions of Federal Legislators Who Received Support From the American College of Obstetricians and Gynecologists, 2012-2022
Source: JAMA Netw Open. 2023 Apr 28;6(4):e2310405. doi: 10.1001/jamanetworkopen.2023.10405 (PMC10148190; doi:10.1001/jamanetworkopen.2023.10405)
Supplement: Supplement 1. — eAppendix. Supplemental Methods eReferences [file jamanetwopen-e2310405-s001.pdf]

Supplemental Online Content

Chhabria K, Raman V, Aghagoli G, Brown BP. Abortion policy positions of federal legislators who received support from the American College of Obstetricians and Gynecologists, 2012-2022. *JAMA Netw Open*. 2023;6(4):e2310405. doi:10.1001/jamanetworkopen.2023.10405

eAppendix. Supplemental Methods
eReferences

This supplemental material has been provided by the authors to give readers additional information about their work.

© 2023 Chhabria K et al. *JAMA Network Open.*

eAppendix. Supplemental Methods

We extracted publicly available campaign contribution data for Ob-GynPAC, for January 1, 2012 through June 30, 2022, from the Federal Election Committee (FEC) website. Committee profiles, including those for lobbyist and registrant PACs, are listed on the FEC website. Funds raised and disbursed by these committees are categorized and coded by the FEC. We collected all processed Ob-GynPAC disbursements made to individual candidates, as well as those made in support of a specific candidate, but not given directly to a campaign (e.g.: for media buys, classified as independent expenditures by the FEC). We filtered out operating expenditures (including bank charges, credit card processing fees, and other non-candidate contributions). We then manually reviewed each contribution line to categorize the recipients of funds (either individual legislators or other PACs/committees).

All legislators who received Ob-GynPAC support and were successfully elected to the House of Representatives or Senate were eligible and included. Candidates who never served in elected office and non-voting members of Congress (e.g., the Representative of the Virgin Islands) were excluded, as they did not directly influence federal policy through votes in Congress. As a sensitivity analysis, we calculated Ob-GynPAC's total expenditures on unsuccessful candidates for House or Senate. These donations totaled $423,213 during our study period.

All disbursements classified as non-federal contributions were excluded, including contributions from Ob-GynPAC to statewide races. Though statewide races are undoubtedly influential in reproductive healthcare policy, we opted to focus on federal officeholders as federal policy affects all United States obstetrician-gynecologists and their patients, and because

© 2023 Chhabria K et al. *JAMA Network Open.*

statewide legislator platforms in the time period examined were heterogeneous, even among legislators with the same national party affiliation.

eReferences

1. The Voter's Self Defense System. Vote Smart. http://votesmart.org. Accessed January 18, 2023.
2. On The Issues: Every Political Leader on Every Issue. https://www.ontheissues.org/default.htm. Accessed January 18, 2023.
3. National Right to Life: Protecting Life in America Since 1968. https://www.nrlc.org/. Accessed January 18, 2023.
4. NARAL Pro-Choice America. https://www.prochoiceamerica.org/. Accessed January 18, 2023.

© 2023 Chhabria K et al. *JAMA Network Open.*
