# Supplementary material for: Abortion Policy Positions of Federal Legislators Who Received Support From the American College of Obstetricians and Gynecologists, 2012-2022
Source: JAMA Netw Open. 2023 Apr 28;6(4):e2310405. doi: 10.1001/jamanetworkopen.2023.10405 (PMC10148190; doi:10.1001/jamanetworkopen.2023.10405)
Supplement: Supplement 2. — Data Sharing Statement [file jamanetwopen-e2310405-s002.pdf]

Data Sharing Statement

Chhabria. Abortion Policy Positions of Federal Legislators Who Received Support From the American College of Obstetricians and Gynecologists, 2012-2022. *JAMA Netw Open*. Published April 28, 2023. doi:10.1001/jamanetworkopen.2023.10405

Data
Data available: No

Additional Information
Explanation for why data not available: All data used in this study are already publicly available.
